# Supplementary material for: Machine Learning Accelerates Crystallization for Structure Determination
Source: Angew Chem Int Ed Engl. 2026 May 4;65(25):e1218503. doi: 10.1002/anie.1218503 (PMC13266958; doi:10.1002/anie.1218503)

checkCIF/PLATON report

Structure factors have been supplied for datablock(s) lcz077

THIS REPORT IS FOR GUIDANCE ONLY. IF USED AS PART OF A REVIEW PROCEDURE FOR PUBLICATION, IT SHOULD NOT REPLACE THE EXPERTISE OF AN EXPERIENCED CRYSTALLOGRAPHIC REFEREE.

No syntax errors found. CIF dictionary Interpreting this report

Datablock: lcz077

Bond precision:	C-C = 0.0260 A		Wavelength=1.54184
Cell:	a=7.8391(4)	b=9.6805(4)	c=39.0136(19)
	alpha=90	beta=91.604(4)	gamma=90
Temperature:	100 K		

	Calculated	Reported
Volume	2959.4(2)	2959.4(2)
Space group	P 21/c	P 1 21/c 1
Hall group	-P 2ybc	-P 2ybc
Moiety formula	C15 H3 Ag3 F18 N6, C8 H7 N	C15 H3 Ag3 F18 N6, C8 H7 N
Sum formula	C23 H10 Ag3 F18 N7	C23 H10 Ag3 F18 N7
Mr	1049.99	1049.99
Dx,g cm-3	2.357	2.357
Z	4	4
Mu (mm-1)	17.145	17.145
F000	2000.0	2000.0
F000’	2009.85	
h,k,lmax	9,12,49	9,11,49
Nref	6370	5920
Tmin,Tmax	0.160,0.180	0.156,1.000
Tmin’	0.026	

Correction method= # Reported T Limits: Tmin=0.156 Tmax=1.000
AbsCorr = MULTI-SCAN

Data completeness= 0.929 Theta(max)= 78.545

R(reflections)= 0.1150(5545) wR2(reflections)= 0.2956(5920)

S = 1.172 Npar= 460

The following ALERTS were generated. Each ALERT has the format
test-name_ALERT_alert-type_alert-level.
Click on the hyperlinks for more details of the test.

Alert level A

PLAT971_ALERT_2_A Check Calcd Resid. Dens. 0.93Ang From Ag3 5.02 eA-3

Author Response: This Alert is due to presence of residual density in the presence of heavy metal atom (Ag).

PLAT971_ALERT_2_A Check Calcd Resid. Dens. 0.80Ang From Ag1 4.53 eA-3

Author Response: This Alert is due to presence of residual density in the presence of heavy metal atom (Ag).

PLAT971_ALERT_2_A Check Calcd Resid. Dens. 0.72Ang From Ag3 4.37 eA-3

Author Response: This Alert is due to presence of residual density in the presence of heavy metal atom (Ag).

PLAT971_ALERT_2_A Check Calcd Resid. Dens. 1.06Ang From Ag2 4.19 eA-3

Author Response: This Alert is due to presence of residual density in the presence of heavy metal atom (Ag).

PLAT971_ALERT_2_A Check Calcd Resid. Dens. 1.01Ang From Ag1 3.94 eA-3

Author Response: This Alert is due to presence of residual density in the presence of heavy metal atom (Ag).

PLAT971_ALERT_2_A Check Calcd Resid. Dens. 0.76Ang From Ag2 3.84 eA-3

Author Response: This Alert is due to presence of residual density in the presence of heavy metal atom (Ag).

Alert level B

PLAT342_ALERT_3_B Low Bond Precision on C-C Bonds ....   0.026 Ang.

Author Response: Disordered structure.

PLAT971_ALERT_2_B Check Calcd Resid. Dens. 0.91Ang From Ag3   2.54 eA-3

Author Response: This Alert is due to presence of residual density in the presence of heavy metal atom (Ag).

PLAT972_ALERT_2_B Check Calcd Resid. Dens. 0.74Ang From Ag1   -2.88 eA-3

Author Response: This Alert is due to presence of residual density in the presence of heavy metal atom (Ag).

PLAT972_ALERT_2_B Check Calcd Resid. Dens. 0.76Ang From Ag3   -2.86 eA-3

Author Response: This Alert is due to presence of residual density in the presence of heavy metal atom (Ag).

PLAT972_ALERT_2_B Check Calcd Resid. Dens. 0.85Ang From Ag2   -2.83 eA-3

Author Response: This Alert is due to presence of residual density in the presence of heavy metal atom (Ag).

PLAT972_ALERT_2_B Check Calcd Resid. Dens. 1.39Ang From F009   -2.66 eA-3

Author Response: This Alert is due to presence of residual density in the presence of heavy metal atom (Ag).

PLAT972_ALERT_2_B Check Calcd Resid. Dens. 0.79Ang From Ag3   -2.60 eA-3

Author Response: This Alert is due to presence of residual density in the presence of heavy metal atom (Ag).

PLAT972_ALERT_2_B Check Calcd Resid. Dens. 0.90Ang From Ag1   -2.51 eA-3

Author Response: This Alert is due to presence of residual density in the presence of heavy metal atom (Ag).

Alert level C

```
DIFMX02_ALERT_1_C  The maximum difference density is > 0.1*ZMAX*0.75
            The relevant atom site should be identified.
PLAT082_ALERT_2_C High R1 Value ..................................       0.12 Report
PLAT084_ALERT_3_C High wR2 Value (i.e. > 0.25) ...................       0.30 Report
PLAT097_ALERT_2_C Large Reported Max.  (Positive) Residual Density       4.41 eA-3
PLAT213_ALERT_2_C Atom F00P            has ADP max/min Ratio .....        3.5 prolat
PLAT213_ALERT_2_C Atom F00R            has ADP max/min Ratio .....        3.5 prolat
PLAT234_ALERT_4_C Large Hirshfeld Difference F00J     --C01B     .       0.18 Ang.
PLAT906_ALERT_3_C Large K Value in the Analysis of Variance ......     14.441 Check
PLAT906_ALERT_3_C Large K Value in the Analysis of Variance ......      3.795 Check
PLAT911_ALERT_3_C Missing FCF Refl Between Thmin & STh/L=    0.600         68 Report
                1  0  0,   9  2  0,   8  6  0,   1  1  1,   9  2  1,  -8  6  1,
                8  6  1,   2  5  2,  -8  6  2,   8  6  2,   2  5  3,  -8  6  3,
                8  6  3,   1  0  4,   9  3  4,  -8  6  4,   9  3  5,  -8  6  5,
                9  0  6,   9  1  6,  -1  6  6,   9  1  7,  -1  6  7,   9  0  8,
                8  2  8,   8  4  8,   5  1  9,   8  2  9,   8  4  9,   1  5  9,
                   (   38 More Missing: see the .ckf listing file)
PLAT971_ALERT_2_C Check Calcd Resid. Dens.  1.10Ang From Ag1             2.46 eA-3
```

Author Response: This Alert is due to presence of residual density in the presence of heavy metal atom (Ag).

```
PLAT971_ALERT_2_C Check Calcd Resid. Dens.  1.05Ang From Ag1             2.35 eA-3
```

Author Response: This Alert is due to presence of residual density in the presence of heavy metal atom (Ag).

```
PLAT971_ALERT_2_C Check Calcd Resid. Dens.  1.07Ang From Ag2             2.34 eA-3
```

Author Response: This Alert is due to presence of residual density in the presence of heavy metal atom (Ag).

```
PLAT971_ALERT_2_C Check Calcd Resid. Dens.  0.96Ang From Ag1             2.12 eA-3
```

Author Response: This Alert is due to presence of residual density in the presence of heavy metal atom (Ag).

```
PLAT971_ALERT_2_C Check Calcd Resid. Dens.  0.89Ang From N2              2.04 eA-3
```

Author Response: This Alert is due to presence of residual density in the presence of heavy metal atom (Ag).

```
PLAT971_ALERT_2_C Check Calcd Resid. Dens.  1.30Ang From Ag2             2.04 eA-3
```

Author Response: This Alert is due to presence of residual density in the presence of heavy metal atom (Ag).

```
PLAT971_ALERT_2_C Check Calcd Resid. Dens.  1.15Ang From Ag3            1.94 eA-3
```

Author Response: This Alert is due to presence of residual density in the presence of heavy metal atom (Ag).

```
PLAT971_ALERT_2_C Check Calcd Resid. Dens.  1.36Ang From F008           1.94 eA-3
```

Author Response: This Alert is due to presence of residual density in the presence of heavy metal atom (Ag).

```
PLAT971_ALERT_2_C Check Calcd Resid. Dens.  1.21Ang From Ag3            1.91 eA-3
```

Author Response: This Alert is due to presence of residual density in the presence of heavy metal atom (Ag).

```
PLAT971_ALERT_2_C Check Calcd Resid. Dens.  1.29Ang From Ag3            1.90 eA-3
```

Author Response: This Alert is due to presence of residual density in the presence of heavy metal atom (Ag).

```
PLAT971_ALERT_2_C Check Calcd Resid. Dens.  1.21Ang From Ag3            1.86 eA-3
```

Author Response: This Alert is due to presence of residual density in the presence of heavy metal atom (Ag).

```
PLAT971_ALERT_2_C Check Calcd Resid. Dens.  0.98Ang From F00G           1.81 eA-3
```

Author Response: This Alert is due to presence of residual density in the presence of heavy metal atom (Ag).

```
PLAT971_ALERT_2_C Check Calcd Resid. Dens.  1.17Ang From Ag1            1.77 eA-3
```

Author Response: This Alert is due to presence of residual density in the presence of heavy metal atom (Ag).

```
PLAT971_ALERT_2_C Check Calcd Resid. Dens.  1.26Ang From Ag1            1.73 eA-3
```

Author Response: This Alert is due to presence of residual density in the presence of heavy metal atom (Ag).

```
PLAT971_ALERT_2_C Check Calcd Resid. Dens.  1.42Ang From Ag2            1.69 eA-3
```

Author Response: This Alert is due to presence of residual density in the presence of heavy metal atom (Ag).

```
PLAT971_ALERT_2_C Check Calcd Resid. Dens.  1.39Ang From Ag3            1.67 eA-3
```

Author Response: This Alert is due to presence of residual density in the presence of heavy metal atom (Ag).

```
PLAT971_ALERT_2_C Check Calcd Resid. Dens.  0.78Ang From C00Y           1.66 eA-3
```

Author Response: This Alert is due to presence of residual density in the presence of heavy metal atom (Ag).

```
PLAT971_ALERT_2_C Check Calcd Resid. Dens.  0.83Ang From Ag2            1.65 eA-3
```

Author Response: This Alert is due to presence of residual density in the presence of heavy metal atom (Ag).

```
PLAT972_ALERT_2_C Check Calcd Resid. Dens.  0.85Ang From Ag1           -2.50 eA-3
```

Author Response: This Alert is due to presence of residual density in the presence of heavy metal atom (Ag).

```
PLAT972_ALERT_2_C Check Calcd Resid. Dens.  2.01Ang From Ag1           -2.35 eA-3
```

Author Response: This Alert is due to presence of residual density in the presence of heavy metal atom (Ag).

```
PLAT972_ALERT_2_C Check Calcd Resid. Dens.  1.78Ang From Ag1           -2.34 eA-3
```

Author Response: This Alert is due to presence of residual density in the presence of heavy metal atom (Ag).

```
PLAT972_ALERT_2_C Check Calcd Resid. Dens.  0.82Ang From Ag2           -2.28 eA-3
```

Author Response: This Alert is due to presence of residual density in the presence of heavy metal atom (Ag).

```
PLAT972_ALERT_2_C Check Calcd Resid. Dens.  0.82Ang From Ag1           -2.28 eA-3
```

Author Response: This Alert is due to presence of residual density in the presence of heavy metal atom (Ag).

PLAT972_ALERT_2_C Check Calcd Resid. Dens. 1.10Ang From Ag2 -2.12 eA-3

Author Response: This Alert is due to presence of residual density in the presence of heavy metal atom (Ag).

PLAT972_ALERT_2_C Check Calcd Resid. Dens. 0.96Ang From Ag1 -2.00 eA-3

Author Response: This Alert is due to presence of residual density in the presence of heavy metal atom (Ag).

PLAT972_ALERT_2_C Check Calcd Resid. Dens. 1.11Ang From F00G -1.94 eA-3

Author Response: This Alert is due to presence of residual density in the presence of heavy metal atom (Ag).

PLAT972_ALERT_2_C Check Calcd Resid. Dens. 1.12Ang From N6 -1.88 eA-3

Author Response: This Alert is due to presence of residual density in the presence of heavy metal atom (Ag).

PLAT972_ALERT_2_C Check Calcd Resid. Dens. 0.96Ang From Ag3 -1.87 eA-3

Author Response: This Alert is due to presence of residual density in the presence of heavy metal atom (Ag).

PLAT972_ALERT_2_C Check Calcd Resid. Dens. 1.09Ang From Ag3 -1.86 eA-3

Author Response: This Alert is due to presence of residual density in the presence of heavy metal atom (Ag).

PLAT972_ALERT_2_C Check Calcd Resid. Dens. 1.70Ang From F008 -1.85 eA-3

Author Response: This Alert is due to presence of residual density in the presence of heavy metal atom (Ag).

PLAT972_ALERT_2_C Check Calcd Resid. Dens. 1.97Ang From Ag2 -1.83 eA-3

Author Response: This Alert is due to presence of residual density in the presence of heavy metal atom (Ag).

```
PLAT972_ALERT_2_C Check Calcd Resid. Dens.  1.02Ang From Ag3           -1.83 eA-3
```

Author Response: This Alert is due to presence of residual density in the presence of heavy metal atom (Ag).

```
PLAT972_ALERT_2_C Check Calcd Resid. Dens.  1.51Ang From F00A          -1.79 eA-3
```

Author Response: This Alert is due to presence of residual density in the presence of heavy metal atom (Ag).

```
PLAT972_ALERT_2_C Check Calcd Resid. Dens.  0.99Ang From F00G          -1.78 eA-3
```

Author Response: This Alert is due to presence of residual density in the presence of heavy metal atom (Ag).

```
PLAT972_ALERT_2_C Check Calcd Resid. Dens.  1.76Ang From Ag2           -1.78 eA-3
```

Author Response: This Alert is due to presence of residual density in the presence of heavy metal atom (Ag).

```
PLAT972_ALERT_2_C Check Calcd Resid. Dens.  1.23Ang From N1            -1.77 eA-3
```

Author Response: This Alert is due to presence of residual density in the presence of heavy metal atom (Ag).

```
PLAT972_ALERT_2_C Check Calcd Resid. Dens.  1.68Ang From Ag2           -1.71 eA-3
```

Author Response: This Alert is due to presence of residual density in the presence of heavy metal atom (Ag).

```
PLAT977_ALERT_2_C Check Negative Difference Density on H00Y      .      -0.72 eA-3
PLAT977_ALERT_2_C Check Negative Difference Density on H016      .      -0.49 eA-3
PLAT977_ALERT_2_C Check Negative Difference Density on H018      .      -0.92 eA-3
PLAT977_ALERT_2_C Check Negative Difference Density on H01D      .      -0.33 eA-3
```

Alert level G

```
PLAT002_ALERT_2_G Number of Distance or Angle Restraints on AtSite         24 Note
PLAT003_ALERT_2_G Number of Uiso or U(i,j)  Restrained non-H-Atoms         13 Report
PLAT007_ALERT_5_G Number of Unrefined Donor-H Atoms .............           1 Report
              H016
PLAT083_ALERT_2_G SHELXL Second Parameter in WGHT  Unusually Large     381.41 Why ?
PLAT172_ALERT_4_G The CIF-Embedded .res File Contains DFIX Records         26 Report
PLAT186_ALERT_4_G The CIF-Embedded .res File Contains ISOR Records          3 Report
PLAT242_ALERT_2_G Low    'MainMol' Ueq as Compared to Neighbors of       C010 Check
PLAT242_ALERT_2_G Low    'MainMol' Ueq as Compared to Neighbors of       C011 Check
PLAT242_ALERT_2_G Low    'MainMol' Ueq as Compared to Neighbors of       C012 Check
```

```
PLAT242_ALERT_2_G Low    'MainMol' Ueq as Compared to Neighbors of       C01A Check
PLAT242_ALERT_2_G Low    'MainMol' Ueq as Compared to Neighbors of       C01B Check
PLAT720_ALERT_4_G Number of Unusual/Non-Standard Labels .........          52 Note
              F004    F005    F006    F007    F008    F009    F00A    F00B
              F00C    F00D    F00E    F00F    F00G    F00J    F00N    F00P
              F00Q    F00R    C00S    C00T    C00U    C00V    C00W    C00X
              C00Y    H00Y    C00Z    H00Z    C010    C011    C012    C013
              H013    C014    C015    N016    H016    C017    H017    C018
              H018    C019    H019    C01A    C01B    C01C    C01D    H01D
              C01E    H01E    C01F    H01F
PLAT860_ALERT_3_G Number of Least-Squares Restraints ............         104 Note
PLAT883_ALERT_1_G Absent Datum for _atom_sites_solution_primary ..     Please Do !
PLAT910_ALERT_3_G Missing FCF Reflection(s) Below Theta(Min)[Deg]=       4.71 Note
                0  0  2,   0  0  4,
PLAT912_ALERT_4_G Missing # of FCF Reflections Above STh/L=  0.600        339 Note
PLAT941_ALERT_3_G Average HKL Measurement Multiplicity ..........         2.0 Low
PLAT969_ALERT_5_G The 'Henn et al.' R-Factor-gap value ..........       7.999 Note
              Predicted wR2: Based on SigI**2  3.69 or SHELX Weight 25.23
PLAT978_ALERT_2_G Number C-C Bonds with Positive Residual Density.          1 Info
```

```
   6 ALERT level A = Most likely a serious problem - resolve or explain
   8 ALERT level B = A potentially serious problem, consider carefully
  51 ALERT level C = Check. Ensure it is not caused by an omission or oversight
  19 ALERT level G = General information/check it is not something unexpected

   2 ALERT type 1 CIF construction/syntax error, inconsistent or missing data
  67 ALERT type 2 Indicator that the structure model may be wrong or deficient
   8 ALERT type 3 Indicator that the structure quality may be low
   5 ALERT type 4 Improvement, methodology, query or suggestion
   2 ALERT type 5 Informative message, check
```

It is advisable to attempt to resolve as many as possible of the alerts in all categories. Often the minor alerts point to easily fixed oversights, errors and omissions in your CIF or refinement strategy, so attention to these fine details can be worthwhile. It is up to the individual to critically assess their own results and, if necessary, seek expert advice.

Validation response form

Please find below a validation response form (VRF) that can be filled in and pasted into your CIF.

```
# start Validation Reply Form
_vrf_DIFMX02_lcz077
;
PROBLEM: The maximum difference density is > 0.1*ZMAX*0.75
RESPONSE: ...
;
_vrf_PLAT082_lcz077
;
PROBLEM: High R1 Value ................................       0.12 Report
RESPONSE: ...
;
_vrf_PLAT084_lcz077
;
```

```
PROBLEM: High wR2 Value (i.e. > 0.25) ...................       0.30 Report
RESPONSE: ...
;
_vrf_PLAT097_lcz077
;
PROBLEM: Large Reported Max.  (Positive) Residual Density       4.41 eA-3
RESPONSE: ...
;
_vrf_PLAT213_lcz077
;
PROBLEM: Atom F00P            has ADP max/min Ratio .....        3.5 prolat
RESPONSE: ...
;
_vrf_PLAT234_lcz077
;
PROBLEM: Large Hirshfeld Difference F00J     --C01B     .       0.18 Ang.
RESPONSE: ...
;
_vrf_PLAT906_lcz077
;
PROBLEM: Large K Value in the Analysis of Variance ......     14.441 Check
RESPONSE: ...
;
_vrf_PLAT911_lcz077
;
PROBLEM: Missing FCF Refl Between Thmin & STh/L=    0.600         68 Report
RESPONSE: ...
;
_vrf_PLAT977_lcz077
;
PROBLEM: Check Negative Difference Density on H00Y      .      -0.72 eA-3
RESPONSE: ...
;
# end Validation Reply Form
```

PLATON version of 17/09/2025; check.def file version of 17/09/2025

---

duplicate check

No duplication found

Datablock lcz077 - ellipsoid plot

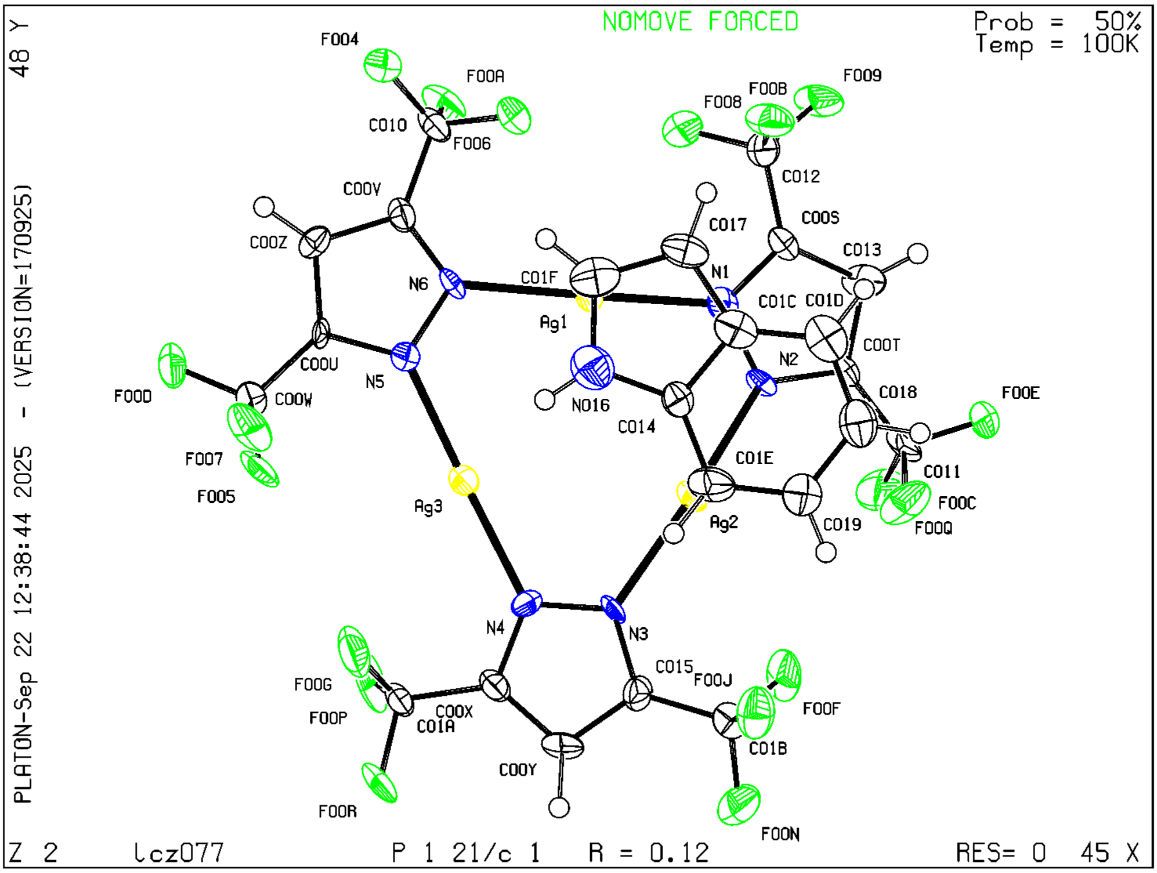

Supplement: Supplementary file 2 — Supporting File 2: anie72462‐sup‐0002‐DataFile.zip. [file ANIE-65-e1218503-s002.zip › supplementary_materials_crystallographic_data submit.zip!/cif_and_checkcif(1-114)/Ag3Pz3_77/Ag3Pz3_77_checkcif.pdf]
